# Discovering topics and trends in biosecurity law research: A machine learning approach

**DOI:** 10.1016/j.onehlt.2024.100964

**Published:** 2024-12-29

**Authors:** Yang Liu

**Affiliations:** Murdoch University, Perth, WA 6150, Australia

**Keywords:** Biosecurity legislation, Topic model, Latent Dirichlet allocation (LDA), Topic distribution

## Abstract

This study employed machine learning techniques, specifically Latent Dirichlet Allocation (LDA), to analyze 559 articles on biosecurity legislation from 1996 to 2023. The LDA model identified nine key research topics, including Agricultural Management and Production, Biosafety and Environmental Impact, Biological Invasion and Regulation, Biosecurity Legislation and Prevention, Agriculture and Environmental Relations, Virus Infection and Governance, Health Risk Assessment and Detection, Disease Prevention and Biotechnology, and Policy Control and Research. The findings reveal significant trends: an increasing focus on Biosecurity Legislation and Prevention and a declining interest in Agricultural Management and Production. Geographically, Australia, Canada, and the United States lead in biosecurity research, exhibiting diverse research topics. Journal-level analysis highlights central topics such as Agricultural Management and Production, Biosecurity Legislation and Prevention, and Health Risk Assessment and Detection. This study's use of LDA reduces subjective bias, providing a more objective analysis of global biosecurity legislation literature. The research underscores the importance of expanding geographical scope, integrating advanced machine learning models, adopting interdisciplinary approaches, and assessing policy impacts to enhance biosecurity strategies globally.

## 1 Introduction

Biosecurity problems are becoming more and more important these days, biosecurity aims to protect humans, animals, and the environment from harmful biological agents through prevention, detection, and response measures (Meyerson and Reaser, 2002). Similarly, biosafety refers specifically to the safety procedures and containment requirements associated with working with certain biological material [15]. While the distinction between biosecurity and biosafety has traditionally been well-defined, recent reports from the World Health Organization (WHO) and the Law Library of Congress suggest that this boundary is increasingly blurred, particularly in the context of emerging biological threats and integrated approaches to governance. This evolving overlap may influence the scope and focus of biosecurity-related research and legislative analysis, highlighting the need for further exploration. Biosecurity is transforming the ways in which public health and environmental protection are managed [10]. Over the years, biosecurity has been applied in areas such as animal health management [11,19], agricultural production [14], food safety [26], pathogen monitoring and control [25], and ecological protection [23]. MacLeod & Spence [13] pointed out that in the context of the sudden disruption of social production by COVID-19, biosecurity technologies have become key to addressing social issues.

At the same time, biosecurity issues have become increasingly prominent in recent years, due to factors such as globalization, urbanization, and climate change, and pose serious threats to human health, social stability, agricultural production and the environment [4]. The COVID-19 pandemic, which caused millions of deaths and trillions of dollars of economic losses since 2020, has drawn a high degree of attention and importance to biosecurity issues worldwide [6]. Biosecurity issues, despite their long history, received little attention until the 1980s, and in the 21st century have increasingly affected social life, food security, the environment and health. The research field of biosecurity legislation has developed over the years, covering many aspects, such as animal husbandry [5], medicine [33], tourism [16], alien species invasion [1] and disease [28].

Various countries have formulated or revised relevant laws and regulations to cope with biosecurity issues and improve their governance capacity and level [20]. The development of biosecurity legislation research exhibits significant differences in content, scope, mode, and effectiveness among different countries [21]. These differences may result in unsatisfactory implementation of biosecurity legislation, or even legal conflicts and disputes [36].

Therefore, this paper conducted research on the biosecurity legislation literature, analyzeed the current situation and differences of biosecurity research in different countries, explored the development trend and improvement direction of global biosecurity legislation research, and provide recommendations for its improvement and coordination.

## 2 Material and methods

This paper employed machine learning methods to extract topics and analyze the distribution of biosecurity legislation research literature to reveal research hotspots and trends in this field. The Latent Dirichlet Allocation (LDA) model was utilized to analyze a combined database of 559 literature titles, abstracts, and keywords from the Web of Science (WoS) database, spanning the years 1996 to 2023. This time span was selected to encompass the key period during which biosecurity legislation gained global attention and witnessed significant development. The analysis focuses on summarizing the topic distribution from three perspectives: year, country, and journal, and exploring the changes over time and across different regions and publications. Yu & Xiang [34] demonstrated the superior performance of the LDA method compared to other methods, particularly in its ability to uncover hidden themes and patterns in large-scale textual datasets, supporting its application in this study.

The methodology was divided into three main modules:

• **Data Collection and Preprocessing:** Literature data were collected from the WoS database by searching for relevant articles. The titles, abstracts, and keywords of these articles were then combined to form a comprehensive database for analysis.
• **Latent Dirichlet Allocation (LDA):** The LDA model was applied to identify latent topics within the combined database, with model parameters optimized for best performance.
• **Exploring Topic Distribution:** The topic distribution was analyzed from multiple perspectives, including temporal (year), geographical (country), and publication source (journal), to uncover trends and patterns.

**Fig. 1:**
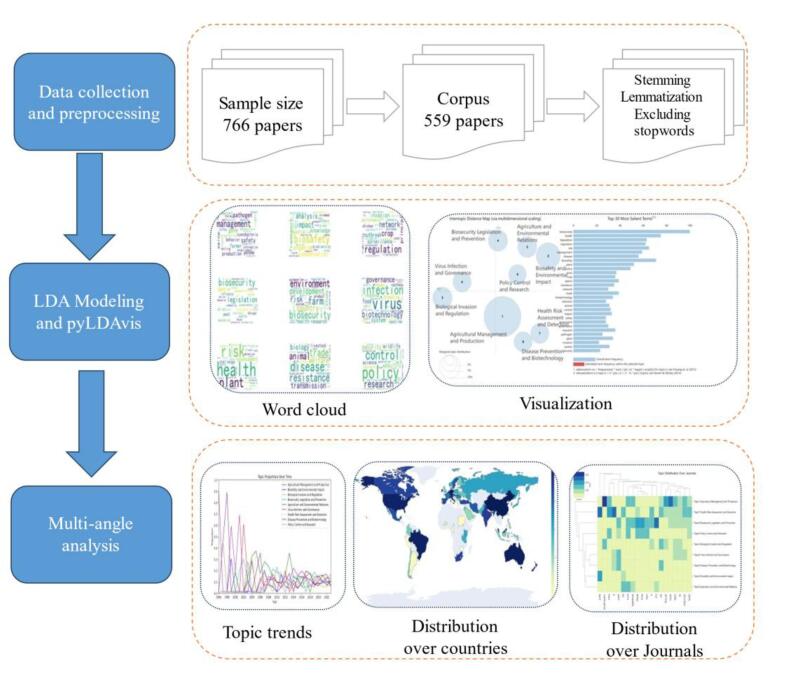
Framework of this research.

Fig. 1. Workflow of the Biosecurity Legislation Research Analysis: This figure illustrates the workflow of the biosecurity legislation research analysis, highlighting the main stages and methods used. The process began with data collection and preprocessing, where a total of 766 papers were initially collected from the Web of Science (WoS) database. After filtering and preprocessing, a corpus of 559 papers was finalized, involving steps such as stemming, lemmatization, and excluding stopwords to prepare the data for analysis. The Latent Dirichlet Allocation (LDA) model was then applied to the preprocessed corpus to identify latent topics within the biosecurity legislation literature. The results of the LDA model were visualized using various methods, including word clouds to display the most frequent terms associated with different topics and topic visualizations showing the distribution and relevance of identified topics. The analysis further explored topic trends over time, the geographical distribution of biosecurity legislation research topics across different countries, and the distribution of topics across various academic journals, providing a comprehensive overview of the research process and findings.

### 2.1 Data collection and preprocessing

As is shown in Fig. 1, the first step of this research was data collection. To collect comprehensive and reliable data, this study used the Web of Science (WoS) as the resource database [12,35]. In order to cover the widest range of biosecurity-related legislation research papers, the keywords searched on WoS included biosecurity law, biosafety law, biosecurity legislation, and biosafety legislation from 1996 to 2023.

This study aggregated literature from the identified sources, extracting details such as the title, abstract, author's address, year of publication, and source [35]. Articles lacking titles or abstracts were omitted. The initial screening yielded a corpus of 559 articles. This study also excised copyright statements from abstracts, amalgamated the title, keywords, and abstract sections, eliminated numbers, punctuation, and stopwords, and excluded words that appeared fewer than five times [35]. To analyze the geographical distribution of publications, the first author's country was inferred from the address details provided in the articles [35]. The Natural Language Toolkit in Python (https://www.nltk.org/) was utilized for the tasks mentioned.

### 2.2 Latent Dirichlet allocation (LDA) modeling

**Fig. 2:**
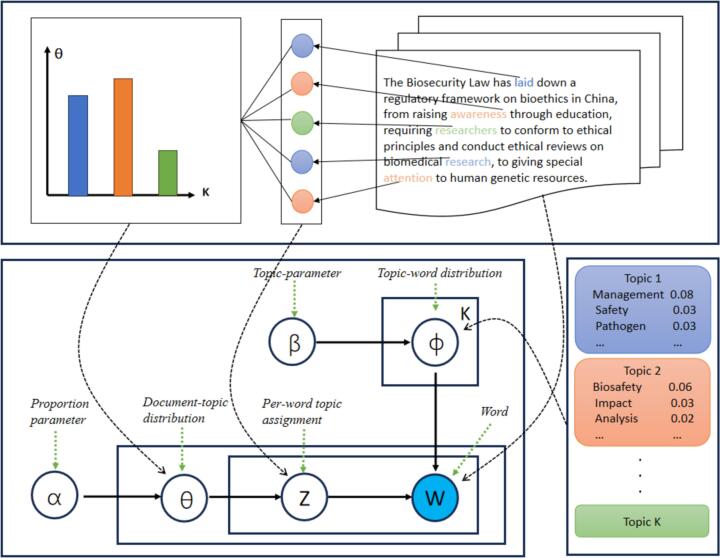
Latent Dirichlet Allocation (LDA) workflow for biosecurity legislation analysis.

Illustrated in Fig. 2 is the generative mechanism of LDA used in This study [3]. Here, *K* denotes the total topics, *M* the document count, and $N_{m}$ the word count within document *m*. Within LDA's framework, each topic is linked to a word distribution, symbolized as$\;\phi_{k}$ . In parallel, every document *m* is linked to a topic distribution, symbolized as $\theta_{m}$. The variable $Z_{\left(m,n\right)}\;$signifies the topic assigned to the nth word in the mth document and $W_{\left(m,n\right)}\;$indicates the actual nth word. The distributions $\;\phi_{k}\;$and $\theta_{m}\;$are smoothed by two hyperparameters, α and β, which the user sets. [2] offers a detailed exploration of this algorithm.

Fig. 2 illustrates the workflow of Latent Dirichlet Allocation (LDA) applied to biosecurity legislation research. The process starts with data preprocessing, where documents are cleaned by stemming, lemmatization, and stopword removal. Key model parameters include *α*, which controls the sparsity of topic distribution per document, and *β*, which controls the sparsity of word distribution per topic. Each document's topic distribution θ is generated from α, while each topic's word distribution ϕ is generated from *β*. For each word in a document, a topic Z is assigned based on θ, and a word W is generated based on ϕ. This iterative process adjusts θ and ϕ to maximize the probability of the observed data. The resulting visualizations include document-topic distributions θ shown in bar charts, and topic-word distributions ϕ shown in word clouds and key term lists.

### 2.3 Experimental setting

This study utilized the gensim library in Python for conducting LDA analysis. It is essential to establish the number of topics before initiating LDA. The determination of an optimal count of nine topics was based on an evaluation of coherence and perplexity metrics, which assess the relation of words and topics. The methodology for identifying the ideal number of topics involved:

• Defining a topic range.
• Implementing topic modeling.
• Calculating coherence and perplexity for each topic.
• Averaging these metrics and conducting a comparative assessment.

The coherence and perplexity outcomes were derived using the following equations:

(1) $$C_{\mathrm{UMass}}=\frac{2}{N\left(N-1\right)}\sum_{i=2}^{N}\sum_{j=1}^{i=1}\log\frac{P\left(w_{i},w_{j}\right)+\epsilon }{P\left(w_{j}\right)}$$

(2) $$\begin{array}{c} \text{perplexity}\left(D\right)=\exp\left(-\frac{\sum_{m=1}^{M}\log P\left(z|m\right)P\left(w|z\right)}{\sum_{m=1}^{M}N_{m}}\right) \end{array}$$

**Fig. 3:**
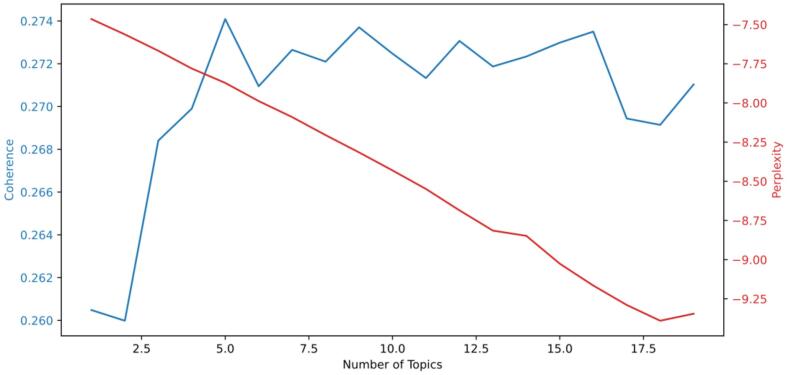
Choosing optimal LDA model: coherence score and perplexity.

In the analysis, the number of topics was determined by evaluating coherence scores, which initially increased and later declined as the topic count rose. A robust coherence score reflects a strong correlation among words, and the optimal topic count is typically selected at the peak or where scores remain consistent [3], which is shown in Fig. 3. To further refine the selection, domain expertise and PyLDAvis (Fig. 5) visualization were employed. The results indicated that nine topics were most appropriate, as the visualization showed no topic overlap, ensuring clear separation and independence. This provides a solid foundation for subsequent multi-perspective analysis.

### 2.4 Exploring the topic distribution from multiple perspectives

The methodology for this aggregation is detailed in 2.4.1, 2.4.2, 2.4.3, 2.4.4, 2.4.5, 2.4.6.

#### 2.4.1 Topic trend in biosecurity legislation

Content from documents published in a particular year was aggregated to reflect the research themes of that year. The topic model used assigns proportions of topics to each document. Consequently, the average of these document-topic proportions for documents within the same year can be considered as the year-topic proportion [35].

(3) $$\begin{array}{c} p_{k}^{y}=\frac{\sum_{m\in Y}p_{\mathrm{mt}}}{q^{y}} \end{array}$$

$p_{k}^{y}$ represents the proportion of topic t in year y.

*m* represents a document published in year y.

*Y* represents the set of all documents published in year y.

$p_{\mathrm{mt}}\;$represents the proportion of topic t in document m.

$\sum_{m\in Y}p_{\mathrm{dk}}$ represents the sum of the proportions of topic t in all documents published in year y.

$q^{y}$ represents the number of documents published in year y.

For example, the time series of topic *k* [$P_{k}^{1996}$, $P_{k}^{1997}$*, …,* $P_{k}^{2024}$]. These time series contain the changing characteristics of the topic distribution and can be employed to discover hot topics.

#### 2.4.2 Topic popularity in biosecurity legislation

This study also applied the criteria outlined by Xiong et al. [32] to assess the popularity of topics. Incorporating popularity of topics allows for the examination of topic evolution over time, providing a more comprehensive understanding of how topics develop and change. This approach helps to overcome limitations such as static analysis and lack of temporal context in traditional models.

(4) $$\begin{array}{c} P^{k}=S_{\mathrm{NP}}^{k}+S_{\mathrm{NTr}}^{k} \end{array}$$

where $P^{k}$represents the popularity score of topics k, which is a comprehensive indicator.

(5) $$\begin{array}{c} S_{\mathrm{NP}}^{k}=\left(P_{A}^{k}-P_{A}^{\min}\right)÷\left(P_{A}^{\max}-P_{A}^{\min}\right) \end{array}$$

$S_{\mathrm{NP}}^{k}$represents the normalized probability score, where $P_{A}^{k}$ is the average topic proportion of topic k.

(6) $$\begin{array}{c} S_{\mathrm{NTr}}^{k}=\left(S_{\mathrm{Tr}}^{k}-S_{\mathrm{Tr}}^{\min}\right)÷\left(S_{\mathrm{Tr}}^{\max}-S_{\mathrm{Tr}}^{\min}\right) \end{array}$$

$S_{\mathrm{NTr}}^{k}\;$is the normalized topic trend score.

(7) $$\begin{array}{c} S_{\mathrm{Tr}}^{k}=\frac{\sum_{2014}^{2023}\gamma_{k}^{y}}{\sum_{y=1996}^{2006}\gamma_{k}^{y}} \end{array}$$

$S_{\mathrm{Tr}}^{k}\;$is the topic trend score.

The algorithms used in this approach are designed to provide a more nuanced and comprehensive understanding of topic popularity. Traditional models often fail to account for temporal dynamics, which can lead to a skewed understanding of a topic's relevance. The dynamic topic model integrates data across multiple years, using algorithms that calculate mean probabilities and trend scores to reflect long-term trends and patterns. This method not only highlights the current popularity of topics but also tracks their evolution, providing a richer and more accurate picture of their significance over time.

#### 2.4.3 Topics distribution over countries/regions

For each country/region c, the proportion of topic k is denoted as$\;\gamma_{k}^{c}$:

(8) $$\begin{array}{c} \gamma_{k}^{c}=\frac{\sum_{d\in c}\gamma_{\mathrm{dk}}}{n^{c}} \end{array}$$

The algorithm used here calculates the cumulative proportion of a specific topic k across all documents from a given country or region c. This is achieved by summing up the topic proportions $\gamma_{\mathrm{dk}}$ for each document d associated with that country/region. The total number of documents $n^{c}$ from that country/region is then used to normalize this sum, providing a proportionate measure of the topic's prevalence within that specific geographic context. This approach addresses potential biases that may arise from considering only the absolute number of documents, ensuring that the calculated proportions are reflective of the relative focus on each topic within the country's or region's research output. Additionally, by attributing research to the country/region of the first author, the method maintains a consistent and practical approach to determining the geographic origin of the research.

This study defined $\gamma^{c,y}$, as the country topic distribution over time. And the representation of the proportion of topic k in year y for each country/region c is expressed as$\;\gamma_{k}^{c,y}$:

(9) $$\begin{array}{c} \gamma_{k}^{c,y}=\frac{\sum_{d\in c\cap d\in y}\gamma_{\mathrm{dk}}}{n^{c,y}} \end{array}$$

In this context, $\sum_{d\in c\cap d\in y}\gamma_{\mathrm{dk}}\;$signifies the cumulative proportion of the $k^{\mathrm{th}}\;$topic in documents from a specific country/region c during year y, and $n^{c,y}\;$denotes the total number of documents published in country/region c during year y.

#### 2.4.4 Hot and cold topics over countries/regions

This article conducted a linear regression analysis on the time-series data of topic distributions. Upon completion of the analysis, a linear equation related to time was derived for each of the nine topics in every country. An aggregation equation, denoted as Eq. (9), compiled the topic distributions per country over various periods. This equation facilitated the integration of research themes from diverse countries, enhancing the visibility of evolving trends in topic distribution, including subtle shifts. A linear regression analysis on the topic distribution time series, utilizing Eq. (10), was then applied to capture the temporal dynamics of topic distribution for each nation. The least squares method was also employed for significance testing based on the linear regression outcomes. Topics that demonstrated significant upward or downward trends in topic proportion across publication sources were identified through linear regression analysis. Conversely, topics with non-significant linear regression outcomes (*p* ≤ 0.05) were omitted.

(10) $$\begin{array}{c} \gamma_{k}^{c,y}=a*y+b \end{array}$$

Where $\gamma_{k}^{c,y}\;$is as defined by Eq. (9). The proportion of topic k in year y for each country c and a and b are the slope and intercept of the equation, respectively.

#### 2.4.5 Information entropy of each country

The information entropy of a country, $e^{c}$, is defined to measure the breadth of the topics covered by its papers [18].

(11) $$\begin{array}{c} e^{c}=-\sum_{k}^{k=1}\gamma_{k}^{c}\ln\left(\gamma_{k}^{c}\right) \end{array}$$

The algorithm used to calculate the information entropy, $e^{c}$, assessed the diversity of research topics within a country's scholarly output. Information entropy is a concept borrowed from information theory, which quantifies the uncertainty or diversity in a set of probabilities. Information entropy indicates that the more disordered the dataset within a system, the greater the entropy; conversely, the more ordered the dataset, the smaller the entropy. In this context, $\gamma_{k}^{c}$  represents the proportion of documents from country ccc that pertain to topic k. The formula for information entropy $e^{c}$ sums the product of each topic's proportion and the natural logarithm of that proportion across all topics. This sum is then multiplied by −1 to ensure a non-negative result. A higher $e^{c}$ value signifies a more diverse set of research topics, indicating that the country's research output is spread across a wide array of topics. Conversely, a lower $e^{c}$ value suggests that the country's research is concentrated in a narrower range of topics. This measure provided valuable insights into the thematic diversity of a country's research contributions, helping to identify areas of specialization and breadth in their academic endeavors.

#### 2.4.6 Topics distribution over publication source

The content of each publication source is represented by the topics it covers. Therefore, the process to ascertain the proportion of each topic linked with a specific publication source adhered to the methodology detailed in section 2.4.3. For every publication source s, the distribution of topics k can be represented as follows:

(12) $$\begin{array}{c} \gamma_{k}^{s}=\frac{\sum_{d\in s}\gamma_{\mathrm{dk}}}{n^{s}} \end{array}$$

In this equation, the term $\sum_{d\in s}\gamma_{\mathrm{dk}}$ signifies the aggregate of the $k^{\mathrm{th}}$ topic proportions issued by the publication source “s “, while $n^{s}\;$indicates the total count of documents released by that source.

The term $\gamma^{s,y}$ reflects the temporal changes in topic distribution within a particular country. The proportion of topic k in year y for each publication source s is denoted as $\gamma_{k}^{c,y}$, which shows the pattern of topic-specific spread among various publication sources in the country over time.

(13) $$\begin{array}{c} \gamma_{k}^{s,y}=\frac{\sum_{d\in s\cap d\in y}\gamma_{\mathrm{dk}}}{n^{s,y}} \end{array}$$

In this context, the summation $\sum_{d\in s\cap d\in y}\gamma_{\mathrm{dk}}\;$represents the aggregate proportion of the $k^{\mathrm{th}}$ topic within documents originating from a particular publication source s during the year y. Meanwhile, $n^{s,y}$ signifies the total count of documents published in country/regions during a year.

The equations used in this section were adapted from existing methodologies in topic modeling and bibliometric analysis [32,35]. By using these established methods, this study aimed to provide a robust analysis of topic trends and distributions.

## 3 Results

### 3.1 Discovering the topics

**Fig. 4:**
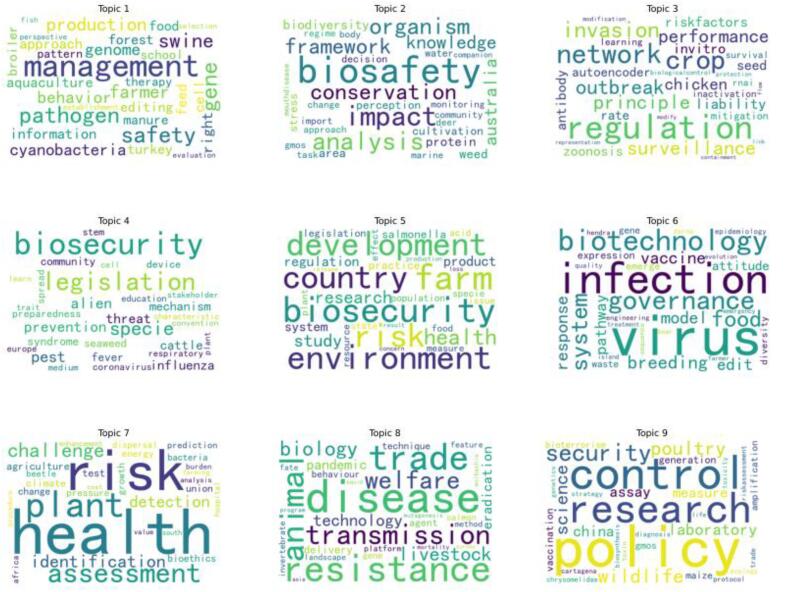
Word cloud diagrams for the nine topics after topic modeling. The figure displays word clouds representing the top keywords for nine different topics identified through topic modeling of biosecurity-related research papers. Each word cloud highlights the most significant terms associated with a specific topic, with larger words indicating higher relevance or frequency within that topic. The topics are labeled from 1 to 9.

This study effectively pinpointed nine core topics within biosecurity legislation and laws using LDA topic modeling. Fig. 4 presents concise thematic labels for these topics. This diagram graphically displays the top 30 words, each sized according to the magnitude of its posterior probability, illustrating their relative importance.

The selected words were integral to this study of biosecurity legislation research, serving to delineate nine distinct topics that represent subfields within biosecurity legislation research. Each topic was defined by its semantic associations. For instance, the topic Agricultural Management and Production incorporates terms like management, safety, pathogen, gene, swine, production, and farmer, which is shown in Table 1.

**Table 1:** Related words and meaning of the nine topics after topic modeling. The first column represents the topic number, the second column is the topic name, and the third column contains the keywords corresponding to the topic.

Topic	Theme	Keywords
1	Agricultural Management and Production	management, safety, pathogen, gene, swine, production, farmer, behavior, genome, cyanobacteria
2	Biosafety and Environmental Impact	biosafety, impact, analysis, organism, conservation, framework, knowledge, Australia, biodiversity, weed,
3	Biological Invasion and Regulation	regulation, network, crop, invasion, outbreak, principle, surveillance, performance, chicken, liability
4	Biosecurity Legislation and Prevention	biosecurity, legislation, specie, pest, alien, prevention, threat, influenza, cattle, mechanism
5	Agriculture and Environmental Relations	biosecurity, farm, environment, development, country, risk, health, research, study, regulation
6	Virus Infection and Governance	virus, infection, biotechnology, governance, system, food, breeding, vaccine, model, response
7	Health Risk Assessment and Detection	health, risk, plant, assessment, challenge, identification, detection, agriculture, test, climate
8	Disease Prevention and Biotechnology	disease, resistance, trade, animal, transmission, welfare, biology, livestock, technology, pandemic
9	Policy Control and Research	policy, control, research, security, poultry, wildlife, science, laboratory, China, assay

### 3.2 Topic visualization

**Fig. 5:**
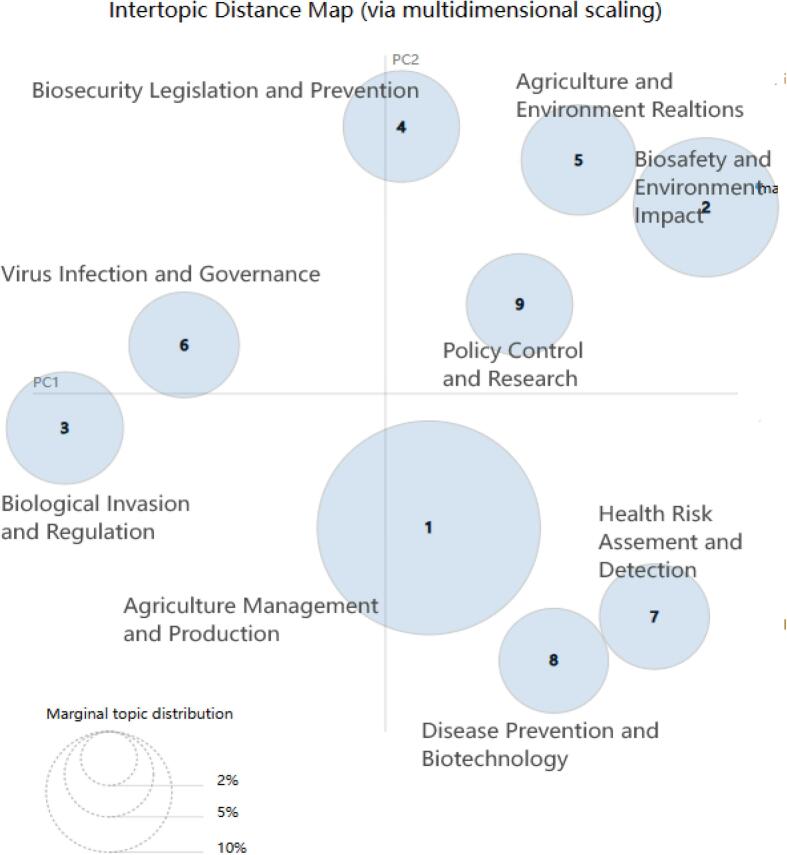
Inter-topic distance map of the Latent Dirichlet Allocation (LDA) topics. The circles on the left represent the nine topics obtained through text mining, which are Agricultural Management and Production, Biosafety and Environmental Impact, Biological Invasion and Regulation, Biosecurity Legislation and Prevention, Agriculture and Environmental Relations, Virus Infection and Governance, Health Risk Assessment and Detection, Disease Prevention and Biotechnology, Policy Control and Research. The size of the circle represents the number of topics. The larger the circle, the more topics there are. The distance between the circles represents the similarity between the topics. The closer the distance between the circles, the higher the similarity. Multidimensional scaling (MDS) is a statistical technique used to visualize the level of similarity of individual cases of a dataset. In this context, MDS helps in creating a spatial representation of the topics based on their pairwise similarities. This allows for an intuitive understanding of how closely related different topics are in a lower-dimensional space.

In Fig. 5, the distances between the centroids of the circles indicate the interconnectedness and correspondence among topics, shedding light on their relationships. Additionally, the size of each circle corresponds to the topic's significance within the wider body of literature. Furthermore, a bar chart displaying the 30 most salient terms in the dataset was utilized to provide deeper insight into the prevailing themes and concepts. The size of the circles in the image represents the amount of content for each topic. The larger the circle, the more content the topic contains. Thus, it is evident that the topic Agricultural Management and Production has the highest number of keywords, indicating that it comprises the largest share. Additionally, the distances between the circles reveal the similarities between topics, the closer the circles, the higher the similarity, and the further apart, the lower the similarity. For example, the proximity between Health Risk Assessment and Detection and Disease Prevention and Biotechnology suggests a high degree of similarity between these two topics.

### 3.3 Topic trend in biosecurity legislation research and topic popularity

**Fig. 6:**
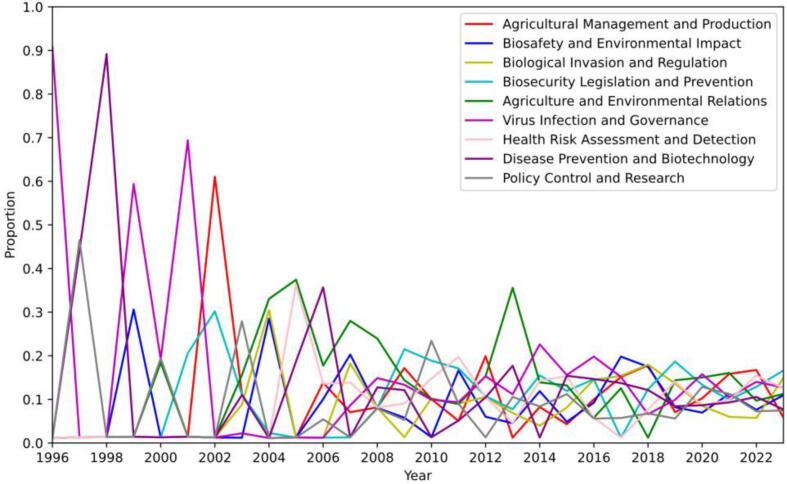
This represents the trend line chart of the development of themes. The nine colors represent nine different themes. The x-axis represents the years, while the y-axis shows the proportion of topics. Each of the nine themes is represented by a distinct color, as indicated in the top-right corner of the figure. The higher the position of the line, the higher the proportion of that topic in that year.

Fig. 6 shows the yearly distribution of topics and subfields, and the relative prevalence of nine topics over time. The line chart in Fig. 6 reveals an intriguing temporal evolution. Virus Infection and Governance dominated the distribution in 1996, accounting for over 90 % of the published proportions. Virus Infection and Governance remained dominant in 1999 and 2001, representing over half of the discourse. However, Virus Infection and Governance prominence declined from 2002, fluctuating in proportion. It resurged between 2014 and 2016, but its prevalence stabilized afterwards, becoming more similar to other topics. Agriculture Management and Production was another noteworthy theme that dominated in 2002. However, its growth trajectory plateaued in recent years. It claimed the largest share in 2022, but its significance dropped to the lowest proportion among the topics in 2023. In contrast, Biosecurity Legislation and Prevention increased significantly since 2000, reaching a notable peak in 2002. The large-scale fluctuations observed during the early years can likely be attributed to the lower number of publications at that time, resulting in higher variability. As the number of publications increased in later years, the trends became more stable. Except for a peak in 2009, its trajectory remained relatively stable. This topic consistently received considerable attention and became the leading theme in 2023. Biological Invasion and Regulation also drew attention as it surged to second place in 2023, indicating a discernible upward trend.

To ascertain the most prominent topics, it was insufficient to simply observe changes in topic distribution over time. Some topics might exhibit a lower proportion yet still maintain a high distribution probability. Consequently, Eqs. (4), (5), (6), (7) are utilized to evaluate the popularity of topics within biosecurity legislation [32]. Table 2 displays the rankings and values of three key indicators for the nine topics. These indicators include: $S_{\mathrm{NP}}^{k}$, which quantifies the novelty of topic k by comparing its frequency in the most recent year's literature against its historical frequency. $S_{\mathrm{NTr}}^{k}$, which tracks the trend of topic k by measuring its frequency in the last year relative to the previous year. $P^{k}$, which represents the comprehensive score of topics k, derived from the weighted average of its novelty and trend scores.

**Table 2:** Comprehensive popularity ranking of topics. $S_{\mathrm{NP}}^{k}$ represents the novelty of the topic: the higher the score, the more novel the topic. $S_{\mathrm{NTr}}^{k}$ represents the trend of the topic. The larger the number, the more the topic is on an upward trend. $P^{k}$ is the combination of these two values. In the table, the first column is the ranking of the topics based on the overall score, and the second column is the topic name. Among the nine topics, the highest overall score was for Biosecurity Legislation and Prevention (T4), with a score of 1.2. The lowest score was for Disease Prevention and Biotechnology (T8), with a score of 0.2.

Rank	Topic	$S_{\mathrm{NP}}^{k}$	$S_{\mathrm{NTr}}^{k}$	$P^{k}$
1	Biosecurity Legislation and Prevention (T4)	0.21	1	1.21
2	Biosafety and Environmental Impact (T2)	0.34	0.33	0.67
3	Agriculture and Environmental Relations (T5)	0.12	0.47	0.59
4	Biological Invasion and Regulation (T3)	0.21	0.37	0.58
5	Health Risk Assessment and Detection (T7)	0.36	0.16	0.52
6	Virus Infection and Governance (T6)	0.25	0.24	0.49
7	Agricultural Management and Production (T1)	0.15	0.19	0.34
8	Policy Control and Research (T9)	0.17	0.07	0.24
9	Disease Prevention and Biotechnology (T8)	0.20	0	0.20

The distribution characteristics of the data show that most topics have a low comprehensive score, and only a few have a high score, resulting in an exponential distribution of $P^{k}$ values. Current hot topics, such as Biosecurity Legislation and Prevention, Biosafety and Environmental Impact, Agriculture and Environmental Relations, have a high $S_{\mathrm{NTr}}^{k}$, indicating a significant increase in their frequency in the most recent year's literature. The outdated or stable topics, such as Policy Control and Research, Disease Prevention and Biotechnology, have a low $S_{\mathrm{NTr}}^{k}$, indicating a decrease or no change in their frequency in the most recent year's literature. The emerging or persistent topics, such as Biological Invasion and Regulation, Health Risk Assessment and Detection, Virus Infection and Governance, have a high $S_{\mathrm{NP}}^{k}$ or a balanced $S_{\mathrm{NTr}}^{k}$ and $S_{\mathrm{NP}}^{k}$, indicating a high frequency or a large ratio to the frequency in all previous years' literature.

The most notable topic was Biosecurity Legislation and Prevention, with a comprehensive score of 1.21, significantly higher than other topics. This indicates that the frequency of occurrence of this topic in the literature over the past year is notably higher than in all previous years, reflecting its importance and urgency.

The least attended topic was Disease Prevention and Biotechnology, with a $P^{k}$ score of 0.20, significantly lower than other topics. This indicates that the frequency of occurrence of this topic in the literature over the past year is zero compared to all previous years, reflecting its backwardness and marginalization.

### 3.4 Topic analysis over countries/regions

**Fig. 7:**
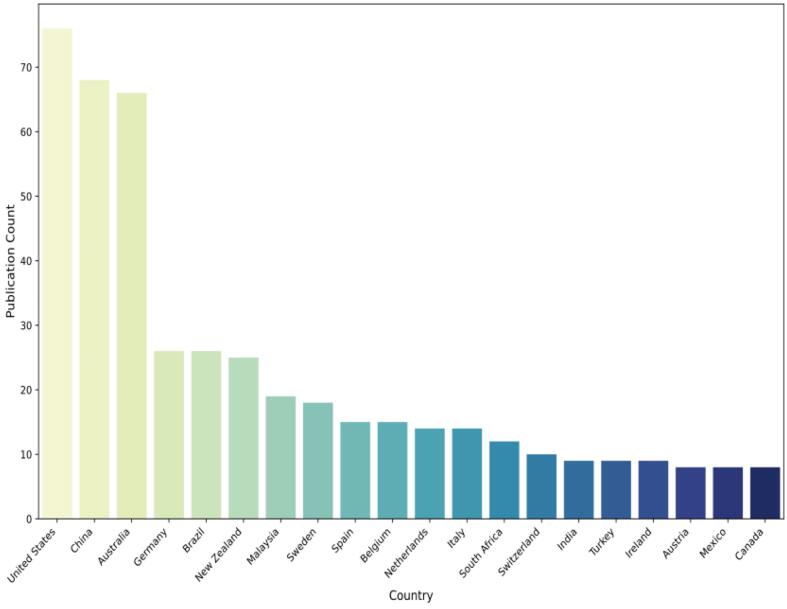
Top publication of the countries.

**Fig. 8:**
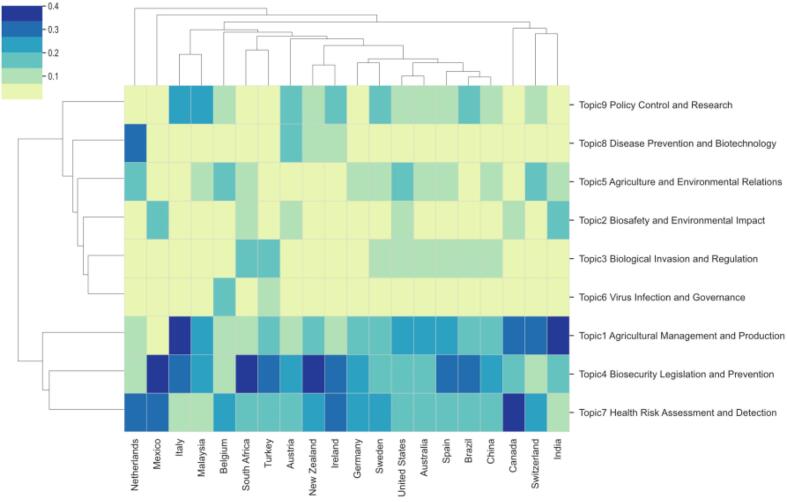
Topic distribution over countries. This heatmap visualizes the distribution of nine topics across various countries, representing the relevance and prevalence of each topic within each country's data. The topics include Agricultural Management and Production, Biosafety and Environmental Impact, Biological Invasion and Regulation, Biosecurity Legislation and Prevention, Agriculture and Environmental Relations, Virus Infection and Governance, Health Risk Assessment and Detection, Disease Prevention and Biotechnology, and Policy Control and Research. The color gradient, ranging from light yellow (low proportion) to dark blue (high proportion), represents the proportion of each topic within the data for each country. Hierarchical clustering groups similar topics and countries together, aiding in identifying patterns and similarities in topic distributions. Countries listed along the x-axis include the Netherlands, Mexico, Italy, Morocco, Malaysia, Spain, Turkey, South Africa, Austria, Hungary, New Zealand, Germany, Sweden, United States, Australia, Brazil, China, Switzerland, and India, while topics are listed along the y-axis. The intensity of the color in each cell indicates the relative importance and frequency of that topic within the corresponding country's data. (For interpretation of the references to color in this figure legend, the reader is referred to the web version of this article.)

To better understand the differences in biosafety and biosecurity research across countries, it would be beneficial to consider the amount and evolution of biosafety and biosecurity legislation over the past 30 years. Variations in legislative focus and frequency may directly influence research priorities and publication trends, reflecting each country's response to specific biosecurity challenges and needs. The analysis concentrated on the top 20 countries by publication volume to explore topic distribution, which is shown in Fig. 7. Fig. 8 features a clustering heatmap, created based on the aggregation method outlined in Eq. (8) [31]. In the heatmap, rows correspond to research publication sources, while columns relate to topics. To measure the distance between countries and topics, Euclidean distance and hierarchical clustering with average linkage were applied [35].

Most countries concentrated their research topics in the field of biosecurity legislation on Agricultural Management and Production, Biosecurity Legislation and Prevention, and Health Risk Assessment and Detection. To illustrate, countries such as Mexico, Italy, Malaysia, South Africa, New Zealand, Ireland, Spain, and Brazil all emphasize Biosecurity Legislation and Prevention. However, subtle differences and unique characteristics existed between these countries. Furthermore, India prioritized Agricultural Management and Production, while Canada gives priority to both Agricultural Management and Production and Health Risk Assessment and Detection. The Netherlands stood alone in focusing on Disease Prevention and Biotechnology. Besides Agricultural Management and Production, Biosecurity Legislation and Prevention, and Health Risk Assessment and Detection, other topics generally received less attention from various countries. Clustering results revealed that many countries grouped into one category lacked a close geographical relationship. This observation suggests that in the field of biosecurity legislation, inter-country cooperation often transcends geographical boundaries.

Fig. 8 illustrates a static analysis, yet the research adopts a dynamic methodology to scrutinize the evolution of topic distributions across countries over time. The prominent and declining topics linked to each publication source are detailed in Table 3.

**Table 3:** The hot and cold topics for all the countries.

Countries	Hot topics	Cold topics
United States	Biosecurity Legislation and Prevention (T4)	Biosafety and Environmental Impact (T2), Disease Prevention and Biotechnology (T8)
New Zealand	Biosecurity Legislation and Prevention (T4), Policy Control and Research (T9)	Biological Invasion and Regulation (T3), Agriculture and Environmental Relations (T5)
Australia	Biosafety and Environmental Impact (T2), Biosecurity Legislation and Prevention (T4), Virus Infection and Governance (T6)	Agricultural Management and Production (T1), Biological Invasion and Regulation (T3)
Canada	Agricultural Management and Production (T1), Virus Infection and Governance (T6), Health Risk Assessment and Detection (T7)	Biosecurity Legislation and Prevention (T4), Policy Control and Research (T9)
Mexico	Agricultural Management and Production (T1)	Biosafety and Environmental Impact (T2), Biological Invasion and Regulation (T3)
Türkiye	Policy Control and Research (T9)	Biological Invasion and Regulation (T3), Virus Infection and Governance (T6)
Spain	Biosafety and Environmental Impact (T2)	Biological Invasion and Regulation (T3), Biosecurity Legislation and Prevention (T4)

If there were no hot topics in a country, they were not shown in this table, which means that the topics of countries not in the table are unpopular topics. This table presents the “hot” and “cold” topics for several countries based on the analysis of topic distributions. Hot topics are highly relevant and frequently discussed within a country's data, while cold topics are less emphasized. The countries analyzed include the United States, New Zealand, Australia, Canada, Mexico, Türkiye, and Spain. For the United States, “Biosecurity Legislation and Prevention (T4)” was a hot topic, whereas “Biosafety and Environmental Impact (T2)” and “Disease Prevention and Biotechnology (T8)” were cold topics. New Zealand highlighted “Biosecurity Legislation and Prevention (T4)” and “Policy Control and Research (T9)” as hot topics, with “Biological Invasion and Regulation (T3)” and “Agriculture and Environmental Relations (T5)” as cold topics. In Australia, the hot topics were “Biosafety and Environmental Impact (T2)”, “Biosecurity Legislation and Prevention (T4)”, and “Virus Infection and Governance (T6)”, while the cold topics were “Agricultural Management and Production (T1)” and “Biological Invasion and Regulation (T3)”. Canada emphasized “Agricultural Management and Production (T1)”, “Virus Infection and Governance (T6)”, and “Health Risk Assessment and Detection (T7)” as hot topics, with “Biosecurity Legislation and Prevention (T4)” and “Policy Control and Research (T9)” being cold topics. Mexico's hot topic was “Agricultural Management and Production (T1)”, and its cold topics are “Biosafety and Environmental Impact (T2)” and “Biological Invasion and Regulation (T3)”. Türkiye's hot topic was “Policy Control and Research (T9)”, with “Biological Invasion and Regulation (T3)” and “Virus Infection and Governance (T6)” as cold topics. Finally, Spain focused on “Biosafety and Environmental Impact (T2)” as a hot topic, while “Biological Invasion and Regulation (T3)” and “Biosecurity Legislation and Prevention (T4)” were cold topics.

**Fig. 9:**
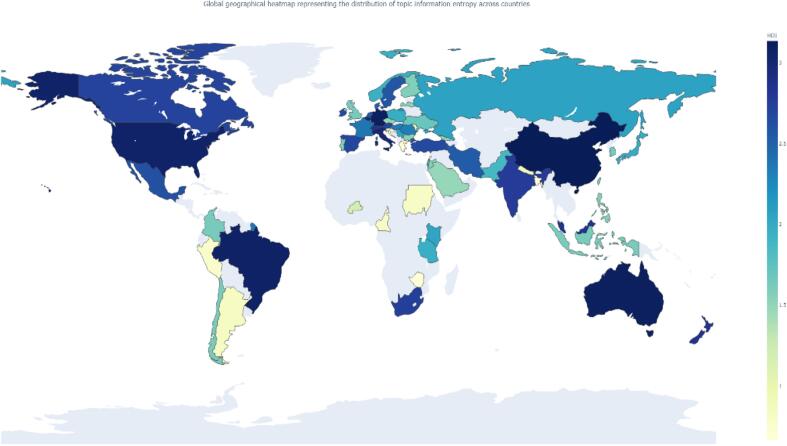
Global geographical heatmap representing the distribution of topic information entropy across countries. The darker the color, the larger the value, which means the wider the research scope of the country. The lower the value, the smaller the research scope of the country. North America, including the United States and Canada, showed a high proportion of topics, indicated by the dark blue shading. European countries, particularly those in Northern and Western Europe, also displayed a high proportion of topics. In Asia, countries such as China and India exhibited significant proportions of topics. South American countries like Brazil and Argentina showed varied proportions, with some areas highlighted in dark blue and others in lighter shades. African countries generally showed lower proportions of topics, indicated by the lighter yellow and green colors. (For interpretation of the references to color in this figure legend, the reader is referred to the web version of this article.)

Using Eq. (11) enabled an in-depth analysis of the range of research topics in each country. A text with a variety of concepts or topics that are evenly distributed may have a higher information entropy. This is indirectly reflected in Fig. 9, where a darker color indicates a richer text in a particular country. The overall distribution of entropy reveals that while most countries have lower entropy values, a select few exhibit higher values. For instance, countries such as Australia, Germany, China, and the United States, all with entropy values exceeding 3.0, demonstrate a relatively broad distribution of research topics. Conversely, countries like Argentina, Bangladesh, Croatia, Greece, Slovenia, and Zimbabwe, all with entropy values below 1.0, show a more concentrated distribution of research topics. The global gradient of entropy distribution reveals that regions such as North America (e.g., the United States and Canada), Western Europe (e.g., the United Kingdom and Spain), Asia (e.g., China, Japan, and South Korea), and Oceania (e.g., Australia) have higher entropy values, suggesting a greater diversity of research topics in these regions. On the other hand, countries in Eastern Europe and Africa, which typically have low entropy, seldom study topics extensively. Additionally, we observed that certain landlocked countries, including Belarus, Uzbekistan, Mongolia, Bolivia, and Paraguay, were absent from the research data we collected. (See Fig. 9.)

### 3.5 Topic analysis over publication source

This component of the research examines topic distribution at the journal level by using Eq. (12), similar to the analysis at the country level in Section 3.4. For the convenience of visualization, this study selected the top twenty journals in terms of publication volume and use abbreviations [35].

**Fig. 10:**
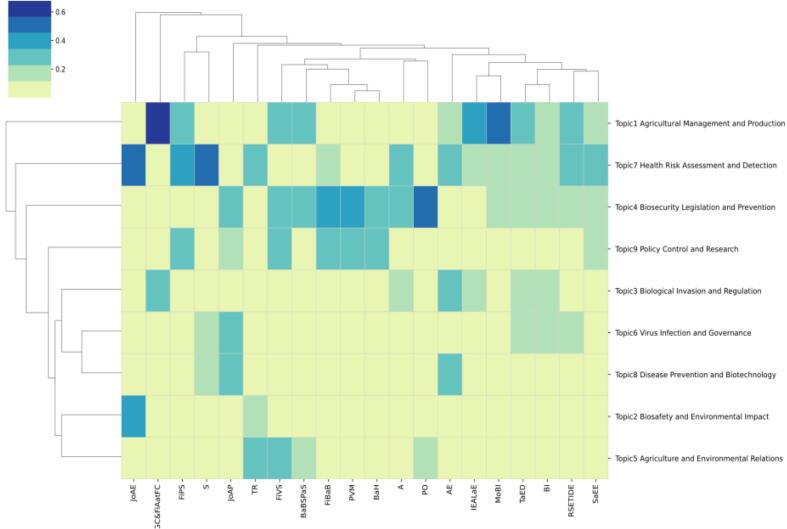
Topic distribution over journals. The rows in the figure represent the subject names, and the columns represent the journal names. The left and top of the figure show hierarchical clustering, where similarities are clustered. This figure illustrates the distribution of biosecurity legislation topics across journals. The heatmap reveals that numerous topics are particularly emphasized by one or multiple publications. For example, Management of Biological Invasions emphasizes Agriculture Management and Production, as does Biosecurity and Bioterrorism-Biodefense Strategy Practice and Science. Additionally, Journal of Applied Ecology, Frontiers in Plant Science, and Sustainability all prioritize Health Risk Assessment and Detection simultaneously. The majority of topics emphasized by the journals were centered around Agricultural Management and Production, Biosecurity Legislation and Prevention, and Health Risk Assessment and Detection. This mirrors the topic distribution at the national level. Simultaneously, these journals exhibited less emphasis on other topics.

Clustering results in Fig. 10 indicate that International Environmental Agreements-Politics Law and Economics and Management of Biological Invasions are clustered together, both concentrating on Agricultural Management and Production. This pattern is also observed in other journals such as Frontiers in Plant Science and Sustainability. Clustering reveals the proximity of conferences based on research content.

Apart from static analysis, dynamic analysis of topic distribution for each journal is imperative. This section employs the linear regression model from section 3.3 to analyze the time series of topic distribution for each journal. Using consistent screening criteria, Table 4 presents the hot and cold topics of each journal.

**Table 4:** This table categorizes the “hot” and “cold” topics for three journals based on the analysis of topic distributions within their published articles. Hot topics were highly relevant and frequently discussed within the journal's articles, while cold topics were less emphasized. The journals analyzed include Biosecurity and Bioterrorism-Biodefense Strategy Practice and Science, Biological Invasions, and Sustainability. Biosecurity and Bioterrorism-Biodefense Strategy Practice and Science highlighted “Biosecurity Legislation and Prevention (T4)” as a hot topic, with “Agricultural Management and Production (T1)” and “Biosafety and Environmental Impact (T2)” as cold topics. Biological Invasions focused on “Virus Infection and Governance (T6)” as a hot topic, while listing “Health Risk Assessment and Detection (T7)” and “Policy Control and Research (T9)” as cold topics. Sustainability emphasized “Biological Invasion and Regulation (T3)” as a hot topic and showed lower emphasis on “Agricultural Management and Production (T1)” and “Virus Infection and Governance (T6)” as cold topics. This legend provides a detailed explanation of the table, offering insights into the thematic focus of each journal and highlighting research trends and focus areas.

Journals	Hot topics	Cold topics
Biosecurity and Bioterrorism-Biodefense Strategy Practice and Science	Biosecurity Legislation and Prevention (T4)	Agricultural Management and Production (T1), Biosafety and Environmental Impact (T2)
Biological Invasions	Virus Infection and Governance (T6)	Health Risk Assessment and Detection (T7), Policy Control and Research (T9)
Sustainability	Biological Invasion and Regulation (T3)	Agricultural Management and Production (T1), Virus Infection and Governance (T6)

Only three journals in the results were statistically significant, while the linear regression results of other topics lack significance. This suggests that within the analyzed dataset, only a handful of topics exhibited notable changes over time, indicating that the distribution of most topics across journals remains relatively stable over the studied time frame, devoid of discernible linear trends.

## 4 Discussion

The exploration of research topics in biosecurity legislation through LDA topic modeling revealed nine distinct themes, each characterized by a set of key terms. The significant increase in attention towards topics like Biosecurity Legislation and Prevention reflects the rising global focus on biosecurity challenges, particularly due to advancements in biotechnology and the increasing frequency of biological threats. This prevalence may also be influenced by high-profile biosecurity incidents, such as the COVID-19 pandemic, which underscored the urgency of robust prevention strategies. Conversely, topics like Agricultural Management and Production have seen a decline, possibly reflecting shifts in funding priorities and the maturation of agricultural biosecurity measures. The differences in prevalent topics across countries are likely shaped by national priorities, economic capacities, and regional threats. For example, developed countries such as the United States and Australia prioritize biosecurity legislation and advanced preventive measures due to their strong policy frameworks and technological capabilities. In contrast, developing regions often focus on agricultural and environmental aspects, driven by economic dependencies and immediate risks in these areas. Understanding these differences highlights the need for tailored biosecurity strategies that address the unique challenges faced by each region. Additionally, sustaining the growth in biosecurity research requires increased funding from governments. This financial support can facilitate interdisciplinary research initiatives and the development of innovative solutions to biosecurity challenges, ensuring robust measures are in place to keep pace with rapid advancements in biotechnology [8].

The analysis of topic distribution across countries revealed a diverse range of research focuses, with the United States, New Zealand, Australia, and Canada showing significant interest in Biosecurity Legislation and Prevention, while countries like India and the Netherlands prioritized Agricultural Management and Production and Disease Prevention and Biotechnology, respectively. This variation reflects differing national priorities and research capacities. To address the global nature of biosecurity threats, it is imperative to promote international collaboration, which involves sharing research findings, harmonizing biosecurity standards, and jointly developing biosecurity policies. Enhanced international collaboration can effectively address cross-border biosecurity challenges [17]. Additionally, policymakers should tailor biosecurity policies to their specific national contexts, considering local threats, resources, and capacities to ensure that measures are relevant and effective. Countries with unique biosecurity challenges should develop specialized policies to address their specific needs [29].

The journal-level analysis revealed that topics such as Agricultural Management and Production, Biosecurity Legislation and Prevention, and Health Risk Assessment and Detection are central to biosecurity research. The clustering of journals based on their research focuses indicated a high degree of similarity in the topics they emphasized, suggesting common research interests and collaborative potentials. To further enhance the impact of biosecurity research, journals should be encouraged to publish more studies on emerging topics, including new biotechnological advances and innovative biosecurity measures, to disseminate critical information and foster academic discussions. Encouraging diverse topic publications can broaden the scope of biosecurity research [30]. Additionally, funding agencies and academic institutions should support interdisciplinary research projects to reflect the interdisciplinary nature of biosecurity. Integrating diverse perspectives and expertise can lead to more comprehensive biosecurity solutions, bridging gaps between different fields and providing holistic strategies [24].

The temporal analysis of topics showed dynamic shifts in research focus over time. For example, Biosecurity Legislation and Prevention has consistently received considerable attention, reflecting its importance in addressing global biosecurity challenges. Conversely, topics like Agricultural Management and Production have seen a decline, possibly due to the maturation of agricultural technologies and shifts in research funding and policy directions. Policymakers should monitor trends in biosecurity research to identify emerging threats and areas needing attention, guiding the allocation of resources and the development of proactive policies. Staying updated with research trends can help in preemptively addressing potential biosecurity issues [9]. Additionally, to prevent knowledge gaps, incentives should be provided for research in areas experiencing a decline, such as Agricultural Management and Production. This can include grants, awards, and recognition for work in these critical areas, motivating researchers to explore less popular but important biosecurity topics [22].

The global distribution of research topics, as indicated by the entropy analysis, revealed that countries with high economic and research power, such as the USA, Australia, and China, exhibit a broad range of research topics. In contrast, countries with lower research capacities tend to focus on more specific areas, indicating a potential gap in global biosecurity research efforts. To address this disparity, international organizations and developed nations should assist in building research capacities in developing countries through funding, training programs, and collaborative research initiatives. Enhancing research capacity globally can help address biosecurity issues more effectively [28] . Additionally, establishing platforms for knowledge transfer between countries with advanced research capabilities and those with limited resources can facilitate exchange programs, joint conferences, and collaborative projects. Such knowledge transfer initiatives can strengthen global biosecurity efforts and ensure that all countries are equipped to handle biosecurity threats [8].

## 5 Conclusion

This study employed Latent Dirichlet Allocation (LDA) to analyze 559 articles on biosecurity legislation from 1996 to 2023, identifying nine key research topics and revealing significant trends. The findings demonstrate an increasing focus on Biosecurity Legislation and Prevention, reflecting the global recognition of the importance of robust biosecurity frameworks. Conversely, a declining interest in Agricultural Management and Production highlights shifts in research priorities and funding. The geographical analysis revealed that countries with high economic and research capacities, such as the USA, Australia, and China, exhibit a broad range of research topics, while countries with lower capacities focus on more specific areas. To enhance global biosecurity efforts, it is essential to promote international collaboration, tailor biosecurity policies to national contexts, and support interdisciplinary research. Increasing funding for biosecurity research and encouraging diverse topic publications can facilitate innovative solutions and address emerging biosecurity threats.

Future research should aim to expand the geographical scope to include more diverse countries, integrate advanced machine learning models for deeper insights, and adopt interdisciplinary approaches to address the complex challenges in biosecurity. Encouraging research institutions to include a broader range of countries in their studies can provide a more global perspective on biosecurity issues, helping to identify unique regional challenges and solutions and enhancing the understanding of global biosecurity dynamics [17]. Promoting the use of advanced machine learning and deep learning models in biosecurity research can uncover deeper insights and more nuanced trends, providing more accurate and comprehensive analyses [7]. Additionally, conducting studies to assess the effectiveness of biosecurity legislation in practice can offer valuable feedback for improving existing policies, ensuring they meet their intended objectives, and bridging the gap between research and real-world applications [27].

## Ethics statement

This study did not involve human participants or animals; therefore, ethical approval was not required.

## Declaration of generative AI in scientific writing

Declaration: After completing the writing of this manuscript, the author, Yang Liu, used ChatGPT-4 solely to improve the language and enhance readability. The tool was not used to generate original content, and the author carefully reviewed and edited the output as necessary. The author takes full responsibility for the content of the published article.

## CRediT authorship contribution statement

**Yang Liu:** Writing – review & editing, Writing – original draft, Visualization, Supervision, Software, Methodology, Investigation, Formal analysis, Conceptualization.

## Declaration of competing interest

The author, Yang Liu, declares that there are no conflicts of interest related to the research, authorship, or publication of this article titled “Discovering topics and trends in biosecurity law research: a machine learning approach”. The research was conducted independently, and no financial support or sponsorship that could influence the results of this work was received.

## Figures and Tables

**Table 1:** Abbreviations of journal names.

Abbreviation	Journal Name
BaBSPaS	Biosecurity and Bioterrorism-Biodefense Strategy Practice and Science
PVM	Preventive Veterinary Medicine
FiBaB	Frontiers in Bioengineering and Biotechnology
BI	Biological Invasions
BaH	Biosafety and Health
A	Animals
TaED	Transboundary and Emerging Diseases
TR	Transgenic Research
IEALaE	International Environmental Agreements-Politics Law and Economics
RSETIDE	Revue Scientifique Et Technique-Office International Des Epizooties
SaEE	Science and Engineering Ethics
S	Sustainability
JoAP	Journal of Applied Phycology
FiVS	Frontiers in Veterinary Science
AE	Austral Entomology
FiPS	Frontiers in Plant Science
MoBI	Management of Biological Invasions
PO	Plos One
JoAE	Journal of Applied Ecology
GCandFiAatFC	Gm Crops and Food-Biotechnology in Agriculture and the Food Chain

## Data Availability

The data used in this study were generated and analyzed solely by the author and are available upon reasonable request.

## Appendix A Appendix

The abbreviation table is as follows:
